# Out of the laboratory, into the field: perspectives on social, ethical and regulatory challenges in UK wildlife research

**DOI:** 10.1098/rstb.2020.0226

**Published:** 2021-08-16

**Authors:** Alexandra Palmer, Beth Greenhough

**Affiliations:** School of Geography and the Environment, Oxford Centre for the Environment, University of Oxford, South Parks Rd, Oxford OX1 3QY, UK

**Keywords:** animal research, animal welfare, ethics, fieldwork, regulation, wildlife

## Abstract

Drawing on insights from qualitative social science research, this paper aims to prompt reflection on social, ethical and regulatory challenges faced by scientists undertaking invasive animal research in the field and propose ways of addressing these challenges to promote good care for animals and environments. In particular, we explore challenges relating to the management of (i) relationships with publics and stakeholders, who may be present at field sites or crucial to research success; (ii) ethical considerations not present in the laboratory, such as the impacts of research on populations and ecosystems; (iii) working under an array of regulations, which may operate in accordance with competing ethical principles or objectives; and (iv) relationships with regulators (especially vets), which may involve disagreements over ethics and expertise, especially because regulators may be more accustomed to overseeing research in the laboratory than the field. We argue that flexibility—at a personal and policy level—and respect for others' expertise emerged as two key ways of negotiating ethical challenges, fostering positive working relationships and promoting good care for individual animals and broader ecosystems. While our analysis focuses on the UK, we propose that many of these lessons are broadly applicable to international contexts.

This article is part of the theme issue ‘Measuring physiology in free-living animals (Part II)’.

## 1. Introduction

Laboratory–field borders have long interested scholars in the humanities and social sciences. Attention has focused in particular on how laboratories and fields were historically constructed in opposition to one another and, in some ways, have competed for scientific legitimacy. While laboratories are often presented as stripped-down ‘placeless places’ that produce knowledge which can be universally applied across contexts, fields promise greater realism at the expense of control [1–5]. Of course, it is widely acknowledged that there is not a firm distinction between laboratories and fields, as exemplified by hybrid spaces such as ‘semifield stations’ and mesocosms, and the incorporation of laboratory-like elements into the field and vice versa such as in ‘natural experiments’ [1,6–8]. Still, laboratory–field comparisons are useful for exploring how spatial and social elements can alter the practice and representation of science [4,5].

In this paper, we draw on this tradition of using insights from the social sciences to consider how science works differently in laboratories versus fields. However, we are less concerned with the legitimacy and kind of scientific knowledge produced in these sites than with the social, ethical and regulatory challenges scientists face in the field compared with the laboratory, and what these challenges mean for the health and well-being of animals and ecosystems. In particular, we show that laboratory–field comparisons are valuable for gaining insight into other key themes in social studies of science and conservation, namely: transparency and openness in animal research [9,10]; affective relationships between researchers and wildlife study subjects [11,12]; conflicts between conservation and animal welfare goals [12–17]; negotiating expertise between professional scientists and other knowledge-holders [18–24]; and the relationship between animal welfare law and good care in practice [25–28]. We explore these ethical, social and regulatory elements of field-based animal research through the lens of work covered by the UK's Animals (Scientific Procedures) Act (1986). This research typically involves activities such as sedation, the collection of blood samples or the attachment of tracking devices to free-ranging animals. Our goal is to prompt reflection among researchers and others involved in this work (e.g. vets, regulators) about the unique challenges posed by field-based animal research, and to propose some ways that these parties might work together to improve care for animals and ecosystems. While we draw on qualitative research with animal research communities in the UK, we propose that many of these lessons are broadly applicable to international contexts.

In particular, we focus on four key challenges faced by researchers working in the field, which relate to the management of (i) relationships with publics and stakeholders, who may be present at field sites or crucial to research success; (ii) ethical considerations not present in the laboratory, such as the impacts of research on populations and ecosystems; (iii) working under an array of regulations, which may operate in accordance with competing ethical principles or objectives; and (iv) relationships with regulators (especially vets), which may involve disagreements over ethics and expertise, especially because regulators are likely to be more accustomed to overseeing research in the laboratory than the field. We conclude by arguing that flexibility and mutual respect emerged as two key ways of managing ethical challenges; fostering good relationships between researchers, stakeholders and regulators; and improving care for individual animals and ecosystems. While these might be difficult to implement in practice, and fundamental differences (e.g. in ethical stances) might prove irresolvable, we propose that where possible researchers and others involved in field-based animal research aspire towards these dispositions in order to improve outcomes for animals and ecosystems under study.

## 2. Methods

This paper draws on qualitative social science research undertaken as part of ‘The Animal Research Nexus’ programme (AnNex: https://animalresearchnexus.org/): a collaborative, interdisciplinary project which aims to highlight historical and emerging challenges in the UK animal research sector, and encourage communication across and beyond the communities of animal research [29]. We draw on one specific strand of the AnNex project focusing on sites classed as Places Other than Licenced Establishments (or POLEs) under the Animals (Scientific Procedures) Act (A[SP]A, 1986), which regulates invasive animal research in the UK. POLEs may include wildlife field sites, fisheries, farms, zoos or veterinary clinics.

The POLEs strand of the AnNex project involved semi-structured interviews with 30 people and 24 lengthy informal conversations with others. Together, 21 (38%) of these conversations focused primarily on wildlife research and 5 (9%) on fisheries. A further 10 (19%) conversations were conducted with named veterinary surgeons (NVSs) and former and current A(SP)A regulators and focused on a broad range of sites. Participant-observation was conducted during visits of 1–2 days to five non-laboratory research projects (three of which involved tracking of free-ranging animals), and during shorter site visits, a wildlife research training course and relevant conferences. Notes derived from participant observation and informal conversations are referred to as ‘field notes’ throughout this paper. This qualitative research was not intended to capture views representative of wildlife researchers and regulators, but rather to explore perspectives in-depth and identify key emerging themes. In addition to this qualitative research, the POLEs strand of AnNex involved running a stakeholder workshop on non-laboratory research [30] and a panel discussion on the regulation of wildlife-focused citizen science [31].

Analysis of interview transcripts, field notes and relevant documents was conducted using the qualitative data analysis software NVivo. A policy of de-contextualization has been adopted due to the sensitive nature of the topic; all names presented in this paper are pseudonyms. We also refer only to the class of animal under study (e.g. mammal, fish and bird) rather than the species, given the small number of UK-based wildlife researchers working on a given species. All interviews were conducted with written consent from participants. Ethical approval for this research was granted by the Central University Research Ethics Committee of the University of Oxford (reference number: SOGE 18A-7).

## 3. Relationships with publics and stakeholders

As social scientists have observed, laboratories tend to be spaces inhabited only by researchers and others involved in research support and animal husbandry; by contrast, fields are potentially used by multiple groups [2,4]. Thus, unlike researchers working in laboratory settings (though see [32] for an exception of patient tours of laboratories), those working with free-ranging or agricultural animals may need to negotiate relationships with members of the public and stakeholders, who may also be present at field sites [33].

Several kinds of encounters with publics and stakeholders may occur at field sites. First, members of the public may come across research activities incidentally, such as while out for a walk. Though these encounters are not necessarily adversarial, they may be disruptive. For example, Annika, whose research involves the use of biologging technologies in fish, noted that she has had curious members of the public come by to ask what her team is doing. Sometimes this happens when the team has been undertaking invasive procedures. Annika reflected that while she always tries to engage with people and explain the research, she would never do this at the expense of the fish: she would always ask people to wait if speaking with them would compromise fish welfare (field notes, 15 April 2019). Wild mammal researcher Geoff described how public encounters had become sufficiently disruptive to the research process that his team moved their work from outdoors to an indoor location in a field station (field notes, 7 September 2018). For these reasons, researchers may be explicitly advised to be as discreet as possible when undertaking research in the field (field notes during training course, 11 March 2019). While this advice might appear to run counter to proposals to make research more transparent, public encounters with field research can pose risks to the process of science and the welfare of animals under study.

Researchers working with animals sometimes perceive a personal risk associated with transparency, the concern being that the public may misunderstand their work or animal rights activists will threaten them [9,10]. This may in some cases be less of a risk for wildlife researchers given that public perceptions of such work are often, as researcher Genevieve put it, ‘Disneyesque’ and shaped by positive portrayals of such work in film and other media (interview 21 June 2019; see [34–36] for further discussion). But the public may conflate wildlife research with practices they object to. For example, deliberate sabotage of traps during the hunting season can occur when anti-hunting activists fail to distinguish between traps set for research purposes and those set by hunters (field notes during training course, 11 March 2019). Some wildlife research is also in itself controversial, such as with animals whose management is subject to debate (e.g. invasive species and badgers). This can pose challenges for researchers if they require permission from members of the public to undertake research—such as landowners, companies, game and fisheries trusts and even local wildlife groups—who may in practice act as gatekeepers to research in a particular area. In some countries, indigenous communities may be added to this list. Where stakeholders take issue with research practices or ethics, conflicts can emerge. For example, a researcher involved in a long-running study of free-ranging mammals explained that recently two farmers had retracted permission for the researchers to work on their land, meaning that the researchers immediately lost access to a subset of their study population (field notes, 20 August 2019).

Disagreements about ethics can also go in the other direction, such that researchers may collaborate with stakeholders with whom they disagree. It is important to recognize that approaches to conservation and animal welfare are not universal within social, cultural and ethnic groups. For example, Western conservation approaches based on preservation and protection may be at odds with indigenous groups' traditional practices and customary hunting rights [37,38], and Western and non-Western approaches to animal welfare and ethics may differ [39–41]. Among our participants, fish researcher Greg's work requires collaborating with anglers, who catch fish for use in his research. Yet Greg admitted to having ‘preconceptions’ about working with anglers, because ‘I want [to work with] people that show high levels of empathy and I don't equate fishing with empathy’. In an effort to recruit anglers who would act as ‘more than human winches'—who would catch fish in a manner sensitive to animal welfare—Greg designed a set of questions aimed at assessing anglers' empathy. He explained that, somewhat to his surprise, some anglers' responses strongly implied empathy and concern for fish welfare. For example, some anglers explained their motivations for participating in Greg's project as ‘I am spellbound by these animals’ or similar. Greg added that through this and other similar projects ‘you kind of see empathy in people build’, meaning that interaction with the research may change stakeholders' ethics (interview, 1 October 2019; see also [42]). This example implies that even if researchers and stakeholders start out thinking that they disagree about fundamental ethics, these disagreements can potentially be resolved if both parties take the time to learn from each other's approaches and find areas of agreement. Still, this may not always be possible, in which case researchers may need to consider whether research should proceed if it requires collaborating with stakeholders whose approaches are, in the researchers' eyes, ethically problematic.

Disagreements between researchers and stakeholders may also relate to personal interests. For example, fish researcher Gavin explained that anglers and fisheries management groups may be reluctant to permit research that involves killing fish, or invasive procedures that could harm the welfare or survival of the fish, since they perceive such work as a threat to their stocks (interview with Gavin, 16 January 2019). The issue is therefore not that fisheries managers object to the killing of fish in general, but that they want to ensure that killing only occurs when it serves their interests. When this occurs, a strategy for building good relationships may be to give something back to stakeholders so they feel the research is to their benefit. For example, fish researcher Annika observed that it makes a big difference to relationships when they visit local angling groups and speak with them about the goals of the research, since this makes people feel that they have a stake in the research and would benefit from its results. This approach responds to calls to rethink science–society relationships to make scientific research and outputs more accessible and directly applicable to problems encountered by the public, as part of a broader commitment to ‘Mode 2’ knowledge production that encourages scientific research to be produced with a high degree of social accountability and reflexivity [21,22].

Annika added that she also draws on anglers' expertise, not only to make them feel included but also because it benefits her research, since anglers often hold detailed knowledge of where fish are located in their local area. This reflects how in field science it is not just professional scientists who may possess the expertise of value to scientific research; various other groups may also acquire knowledge (including technical knowledge) through means other than scientific education, such as everyday work and traditional practices, another key dimension of Mode 2 science [23,24]. This observation is the foundation of the field of ethnobiology, which is premised on the idea that indigenous and local knowledge of the environment is not only valuable in its own right, but can also be usefully employed for managing resources and ecosystems [20]. Similarly, citizen scientists—who may also be present at wildlife field sites through acting as skilled volunteers—may hold considerable expertise on catching and handling animals; collaboration with experienced citizen scientists may therefore allow researchers to substantially speed up data collection and even to learn from their collaborators. An important caveat to such alliances, however, is to recognize that citizen scientists and other volunteers may not hold as much experience as researchers, meaning that training or oversight could be required for successful collaboration. This in turn means volunteers could also personally gain from participating in research [31,43,44]. A key lesson for researchers is therefore that the knowledge of volunteers and other stakeholders should be gauged, acknowledged, and where possible put to use for the benefit of the research, and the humans and animals it involves.

## 4. Controllability and ethics

Social scientists have observed that laboratories are often regarded as ‘placeless places’ in that they are intentionally stripped of unique environmental features in order to generate universally applicable findings. By contrast, fields tend to be heterogeneous and distinctive, with this distinctiveness very often the subject of field research [2–4]. This means that while field research can serve as a more accurate reflection of the real world, it can also (rightly or wrongly) be perceived as less replicable and universally relevant than laboratory-based research [2,45,46]. As summarized by fish researcher Gordon, his field research involves ‘so many uncontrolled variables that it's […] very difficult to manage. But you have a real life, wild situation so you have a kind of a serious reality check’ (interview, 15 January 2019). In field-based animal research specifically, sources of variation may arise from genetically heterogeneous populations (potentially necessitating larger sample sizes than in the laboratory), intraspecific variation in behaviour and physiology, and non-standardized environmental conditions [30,47,48].

The reduced controllability of fields compared with laboratories can have important practical, regulatory, ethical and animal welfare implications. From a practical perspective, field-based research can be physically challenging for researchers, which can in turn affect animals' experiences. For example, AP's visit to Annika's fisheries-based research project occurred during very cold weather and required the researchers to at one point cover their work station (which was set up on the back of a truck) with a tarpaulin. Despite this, the poor weather prompted Annika and the other researchers to compare stories of even more challenging weather conditions in which they had previously worked (field notes, 15–16 April 2019). Furthermore, as researcher Hugh noted, the field researcher's equipment is limited to ‘what's in the van’ (field notes, 29 October 2018). Some exceptions to animal welfare standards are therefore explicitly made for wildlife research in A(SP)A guidance. For example, given that wildlife researchers in the field ‘may not have immediate access to drugs, equipment or experienced staff’ as would be expected in a laboratory, in ‘emergency situations' a method of killing not specified by the A(SP)A might be used instead in order to ensure animal suffering is minimized [49]. In other words, animal care may need to be compromised due to difficult research conditions in the field. However, researcher Hugh argued emphatically that this should not lead wildlife researchers to cut corners on animal care. A balance must therefore be struck in negotiations between researchers and regulators, where the limitations of animal care in the field are acknowledged but not used as an excuse for poor standards.

A second challenge deriving from uncontrollability in the field is that ‘you don't know what you're going to catch’ (interview with Genevieve, 21 June 2019), such that researchers may inadvertently catch non-target animals in traps set for research animals. Various steps can be taken to mitigate this risk; for example, one research group AP visited sets traps for nocturnal animals late in the afternoon or evening to avoid catching diurnal species (field notes, 20 August 2019). However, bycatch may be unavoidable and may result in harm to the bycaught animal, if for example animals are injured as a result of traps being designed for a different species. Non-target species may even need to be killed. As researcher Geoff observed, his days occasionally begin with the unpleasant task of killing grey squirrels (*Sciurus carolinensis*), which are unintentionally caught in his team's traps, as EU invasive alien species regulations (1143/2014) dictate that grey squirrels must be killed if caught (field notes, 13 September 2018). Non-target members of the study species may also be inadvertently caught, such as lactating females, which researchers may wish to avoid disturbing due to the knock-on effects on dependent young (interview with Genevieve; for more on the above, see [47]). Bycatch is therefore one way in which wildlife research can affect the welfare not only of research animals, but also other animals living in the same area. As Geoff's example illustrates, these encounters with bycatch can pose an ethical and emotional challenge for researchers.

The conditions under which animals live in the wild are variable and potentially difficult, which means that researchers may also catch animals that are in poor physical condition. Guidance on the A(SP)A indicates that animals taken from the wild found to be ‘injured or in poor health’ should not be ‘subjected to a regulated procedure unless and until it has been examined by a veterinary surgeon or other competent person; and, unless the Secretary of State has agreed otherwise, action has been taken to minimize the suffering of the animal’ [50]. What this means in practice is that researchers may release captured animals that are in poor condition before undertaking research, to avoid noncompliance with research animal welfare regulation. For example, Gordon explained that his team excludes from their study any fish they catch that look especially thin. However, Gordon also explained that doing so suits his research, which focuses on migration tracking: ‘We wouldn't want to use animals which we thought were in some way giving us poor information because they're already in poor condition’. Furthermore, this approach still comes with risks to animal welfare, with many wildlife researchers observing that capture is usually the most stressful part of research for a wild animal (see also [47,51]). Decisions may become trickier when researchers require animals in poor condition for their study. Greg, for example, explained that his research involves a difficult balance between sampling all of his target animals, which includes those in poor health condition when caught, and avoiding causing excessive suffering by tagging and releasing visibly unwell animals. Thus, he explained, ‘I do not really want to [tag and release an unwell animal] but actually I probably should’. Again, Greg's comment implies an ethical and emotional challenge for wildlife researchers posed by catching unwell animals and speaks to a broader problem across all animal research: that harm to animals should be minimized, but not if doing so invalidates the research and therefore means that animals used in the study suffered for no reason.

Further complicating such assessments is researchers' inability to intervene if animals were to suffer or die after release. Ordinarily under the A(SP)A, the personal licence holder's role involves ‘being responsible for the welfare of the animals you have performed procedures on and ensuring that they are properly monitored and cared for’, including ‘making sure that any animal that is in severe pain or severe distress, which cannot be alleviated, is painlessly killed using an appropriate method’ [50]. In wildlife research, animals can potentially be both living freely in the wild and simultaneously under the controls of the A(SP)A, and therefore technically entitled to monitoring, care and euthanasia if they experience severe pain or distress. For example, this occurs when animals are part of an ongoing research project in which they are trapped and released repeatedly, or when they are released with tracking devices. There is therefore a tension whereby the A(SP)A ordinarily assumes that researchers can carefully control the experiences of their research animals to minimize suffering, but such control is impossible in the field, meaning that animal welfare is often unknown and difficult to control.

For this and various other reasons, research participants regularly complained that the A(SP)A was not written with field research in mind, and in some ways is an awkward fit. Similar views have been expressed by researchers working in the USA, who have proposed that Institutional Animal Care and Use Committees (IACUCs) are not oriented towards field-based animal research, and may fail to comprehend the important differences between research in the laboratory and field [33,52–55]. The idea that animal welfare regulations are an awkward fit for field research may refer not only just to practical matters, but also to ethical principles. For example, many research participants referred to the principle of avoiding perturbation as justification for why they would treat any illnesses or injuries that they had clearly caused (e.g. trap-related injuries), but would avoid euthanizing or treating animals where the problems were due to ‘natural’ causes, as this could alter the functioning of the population and ecosystem under study and thereby pose both a scientific and ethical challenge. As well as reflecting an ethical commitment associated with conservation ethics to minimize human interference in the natural world [12,17], this approach also reflects what anthropologist Matei Candea calls ‘inter-patience’: a seemingly paradoxical relationship between researchers and the wildlife they study in which both parties agree to avoid interfering with each other's lives [11]. Of course, the line between ‘natural’ and ‘unnatural’ ailments and injuries may be subjective and unclear, and several participants acknowledged that they have set the principles of non-perturbation and inter-patience aside when they felt emotionally compelled to intervene to avoid visible animal suffering (see also [12]). For example, researcher Graham acknowledged, ‘I confess I've found lizards with ticks on them and I've pulled them off. But that's wearing my compassionate hat; if I'm wearing my hard-headed ecologist hat I'd say let them go’ (interview, 14 September 2018).

Concerns about perturbation reflect the idea that wildlife researchers must consider not only just the effects of their research on the welfare of individual research subjects, but also on wider populations and ecosystems. For this reason, researcher Geoff suggested that an alternative ethic for wildlife research might simply be to ‘do no harm’ by ensuring that ‘the welfare of the animal is not made worse by what you did’ (interview, 7 September 2018). However, it is unclear when, if ever, this could be achieved given the stress potentially caused even just by the capture of free-ranging animals [47,51]. In a similar vein, researcher Graham argued that assessments of harms to animal welfare in wildlife research should consider whether researchers are ‘stressing [free-ranging animals] more than they would be stressed in the wild’. In other words, researchers may feel that welfare assessments and ethical principles should account for the uncontrollability of the field, the difficult conditions that many free-ranging animals experience in their daily lives and the undesirability of perturbation. Some have also proposed that field research ethics should explicitly consider the risks to populations and ecosystems, and animal welfare harms to non-research animals living in the same area. For example, environmental philosopher Howard Curzer and others have proposed that the 3Rs framework—which is commonly applied to animal research around the world and emphasizes *replacing* animals with other methods of testing, *reducing* the number of animals used in research and *refining* procedures to minimize suffering—should be replaced with 9Rs for wildlife research, which prompt consideration of not only individual animal welfare but also the effects of research on populations and ecosystems [56,57].

This suggestion echoes broader conversations about integrating animal welfare with conservation, such as via a ‘compassionate conservation’ approach, which encourages consideration of both the interests of individual animals and ecosystems in conservation decision-making [15–17]. This proposal is made in light of the fact that individual animals are often harmed for the sake of ecosystem or population health (e.g. non-native or overly abundant animals are often culled), which social scientists have identified as the problem of ‘violent care’ inherent in conservation [13,14]. However, neither 9Rs nor compassionate conservation fully resolves these dilemmas, since while both frameworks encourage attending to the interests of both individual animals and broader populations or ecosystems, they do not specify which interests should prevail where they are in conflict [12]. Field-based animal research therefore perhaps inevitably involves tensions between care for individual animals and ecosystems, which researchers and regulators should thoroughly assess and consider.

## 5. Legal complexities

Field research is often governed by a broader range of laws and regulations than laboratory research. Thus, wildlife researchers in the USA have expressed a feeling of ‘running the permit maze’ [54]. We found a similar sense among many wildlife researchers in the UK; some key laws governing UK wildlife research are described in table 1 (see [31] for the original table on which this is based). For example, if a researcher were to undertake a study of a free-ranging non-native bird species such as the Egyptian goose (*Alopochen aegyptiaca*), they may require licences from the Home Office (which oversees the A[SP]A), a Statutory Nature Conservation Organisation (SNCO, e.g. Natural England, Scottish Natural Heritage, which oversee the Wildlife and Countryside Act [WCA]), the British Trust for Ornithology (BTO) and the Animal and Plant Health Agency (APHA). However, other tagging of free-ranging animals may require no licences at all, depending on whether the species is protected under the WCA and other wildlife laws, and whether the research is determined to meet the criteria for inclusion under the A(SP)A, such as whether it is conducted for a ‘scientific purpose’ and exceeds the threshold of invasiveness as defined in the Act. As we have discussed elsewhere, both science- and invasiveness-based criteria are somewhat flexible and in some cases difficult to assess, with the attachment of tracking devices one example of a complex regulatory area [31,58,59].

**Table 1.** Summary of key laws regulating invasive research with free-ranging animals in the UK. Adapted from Palmer *et al*. [31].

law	regulator	summary
Animals (Scientific Procedures) Act (A(SP)A)	Home Office (HO)	Regulates invasive animal research undertaken for scientific purposes. Does not cover: recognized veterinary, agricultural or animal husbandry practice; capture of wild animals; and ringing/marking if the primary purpose is the identification and it causes only momentary pain or distress.
Wildlife and Countryside Act (WCA)	Statutory Nature Conservation Organisations (SNCOs): Natural England, Scottish Natural Heritage, Natural Resources Wales British Trust for Ornithology (BTO) regulates bird ringing	Regulates disturbance, killing and possession of wildlife. Only certain species are protected under the WCA. There are other species-specific laws, e.g. seals. Capture and handling may require extensive training (e.g. bird ringing under the BTO) but also may require no training or licence (e.g. for the European rabbit [*Oryctolagus cuniculus*], red fox [*Vulpes Vulpes*] and wood mouse [*Apodemus sylvaticus*]).
Animal Welfare Act (AWA)	Enforced by various organizations, e.g. the Department for Environment, Food and Rural Affairs (DEFRA), the Royal Society for the Prevention of Cruelty to Animals (RSPCA)	Prohibits animal cruelty and ensures animal welfare needs are met, for any animal ‘under the control of man’. Can apply to wildlife during capture and handling.
EU regulation on Invasive Alien Species (1143/2014)	The Animal and Plant Health Agency (APHA) issues permits in the UK on behalf of DEFRA, which is the competent authority	Outlines prevention, detection, eradication and management of invasive species across the EU. Requires that certain invasive species be killed if caught (e.g. grey squirrels [*Sciurus carolinensis*]).

The various laws under which field-based researchers work sometimes present different advice or requirements for undertaking the same activity. For instance, researcher Geoff observed that when wildlife vaccination is undertaken for animal health, it falls under DEFRA and is licensed by SNCOs, and involves quite specific requirements for animal care, and treatment frequency and dosage. When wildlife vaccination is undertaken for science, it falls under the A(SP)A, which allows researchers more flexibility in creating and justifying their own animal care and treatment procedures to suit the context. This is in line with the commonly made observation that A(SP)A is a deliberately flexible law, which is one of its key strengths. Laws may also operate in accordance with competing ethical principles or objectives. Several participants highlighted what they perceived as insufficient attention to individual animal welfare in the WCA, with animal welfare advocate Gareth observing that not all trapping of animals is regulated under the WCA, and where trapping is regulated there are not always clear requirements around what kind of traps may be used and how trapping is conducted (interview, 27 February 2019). This may be considered part of a broader international trend in which wild animal welfare is viewed as neglected compared with the welfare of captive animals, in philosophy [15], animal research [48,60], wildlife law [61,62] and conservation [15–17].

By contrast, the A(SP)A features considerable focus on individual animal welfare, as demonstrated by its emphasis on the 3Rs and euthanasia as a tool for minimizing animal suffering. Genevieve related her personal experience of Home Office inspectors (HOIs), who oversee implementation of the A(SP)A, as follows:

I would say personally that they are just focused on your subject animal. We've never had, we've never—I don't think—ever had much comeback on non-targets or on conspecifics or anything like that from the Home Office. So yeah, they are very much targeted on the welfare of that individual.

That said, Genevieve acknowledged that her organization— where many experienced wildlife researchers work—extensively considers ecosystem-level effects in their internal ethical review, which occurs before licence applications are sent to the Home Office. It is therefore possible that inspectors feel that ecosystem-level questions have already been well addressed by Genevieve's research group, and that they would ask more questions in other contexts. Furthermore, wildlife-specific guidance on the A(SP)A, which was released in 2016, explicitly highlights the potential environmental consequences of capturing and removing animals from ecosystems, and emphasizes that research with free-ranging animals should be both ‘humane and environmentally sensitive’ [49]. In Genevieve's opinion, this is a good example of how there are ‘definitely moves for the Home Office to at least understand it [wildlife research] a bit more, which is good’, and efforts to outline how the law works differently in the laboratory and field. At the same time, the application of an explicitly animal welfare-focused law like the A(SP)A to wildlife research could be perceived as helping to address the minimal legal attention paid to wild animal welfare. It was for this reason that animal welfare advocate Gareth proposed that the A(SP)A should be extended to cover wildlife capture when undertaken for science. While he acknowledged that this would lead to inconsistency in the regulation of wildlife capture depending on why it was being done, Gareth argued that it would signal the crucial need to consider animal welfare and justifications for trapping in other contexts.

## 6. Regulatory oversight and expertise

The difficulty of balancing risks to animal welfare and ecosystems is sometimes shown through disagreements between researchers and vets involved in oversight and regulation. HOIs, who are primarily responsible for overseeing research under the A(SP)A, are typically vets by training, although a few are medics (interview with HOI Gail, 15 May 2019). Named veterinary surgeons (NVSs) also play an important role at a local level, being responsible for overseeing animal health and welfare within institutions [63]. As in other countries like the USA, relationships between vets and researchers are therefore important for effective regulation, but potentially characterized by lack of understanding or conflict [33,53].

Disagreements between researchers and vets involved in oversight may derive from disciplinary training and ethical focus. This is demonstrated through perspectives on when to treat or euthanize suffering wild animals involved in research, with vets erring on the side of euthanasia and researchers preferring to avoid this. For instance, NVS Gretchen described a disagreement with a group of wildlife researchers over euthanizing or releasing an unwell animal:

I made very clear under certain circumstances they needed to consider euthanasia. As soon as I mentioned euthanasia, everybody shut down. And I find the concept of, that you'd rather release an animal that is potentially suffering acceptable in comparison to kill it and have no suffering, I find-, it's a completely different approach. (Interview, 25 May 2019)

Similarly, a researcher described disagreeing with an NVS's proposal of euthanizing a nocturnal animal that was behaving unusually by ranging during the day (field notes, 20 August 2019). It is important to stress that in other cases vets and researchers agreed over these matters, with several vets for example observing that it is crucial to avoid perturbation in wildlife research. Still, cases of disagreement imply that researchers and vets involved in research oversight may hold differing opinions on what wildlife research ethics ought to involve, with vets tending to focus more on individual animal welfare, and wildlife researchers more concerned with ecosystem-level impacts and avoiding perturbation.

Disagreements between researchers and vets may also arise from perceived differences in expertise, especially in relation to fieldwork. As summarized by researcher Genevieve,

I think a lot of the inspectors come from a lab animal background, so actually understanding this [field research] is quite difficult for them. I don't mean that to sound patronising but I mean I think if you've not experienced doing this kind of work it's really difficult for them to understand the difficulties behind it.

Some HOIs develop a degree of specialism throughout their careers, with a small team within the Home Office specializing in wildlife research (interview with HOI Gail, 15 May 2019); similarly, some NVSs possess expertise in wildlife medicine. However, one such NVS, who has considerable experience with wildlife research, described himself as a ‘rare beast’ in acknowledgement of his unusual skillset (interview with Guy, 8 April 2019). A researcher who works in South Asia similarly noted that the primary vet involved in overseeing her research lacks experience with wildlife (including her study species), implying that this issue could extend to multiple international contexts (interview with Caroline, 11 August 2020). Thus, researchers sometimes described vets' suggestions as odd or ill-informed. For example, Geoff described an HOI's efforts to replace the homemade tables at Geoff's field station (on which samples from sedated animals were collected) with more hygienic stainless-steel benches. However, Geoff objected that due to the lack of heating in the field station, during winter metal benches would be freezing cold. He additionally rejected the inspector's suggestion of placing absorbent pads under the animals to keep them warm, as animals could carry disease and urinate on the pads, thereby generating a large amount of biohazardous waste (interview, 7 September 2019). In short, strategies for improving animal welfare and reducing risks (e.g. of biohazards) in the laboratory do not necessarily work in the field, and researchers with extensive fieldwork experience may be in the best position to identify when this is the case.

Geoff added that the potential for misunderstandings about the realities of fieldwork can be exacerbated because ‘[w]e don't get inspected as often as a laboratory would’. Other participants made similar observations, pointing to the difficulty of arranging for HOIs and NVSs to visit field sites given the short duration of fieldwork seasons, remote locations of field sites, variable and condition-dependent fieldwork schedules, and limited time and resources available to vets. In particular, Geoff noted that his current HOI has never visited his field site in person. For this reason, Geoff suggested, ‘I don't think they understand in real terms what goes on. I don't think they've pictured what we're doing quite the way that we do it. […] Because they've not physically visited the site’. Geoff here suggests that the unique feel of a field space, which shapes relationships between humans and animals [6], needs to be experienced first-hand for field research to be fully understood. Thus, misunderstandings may be exacerbated by vets' limited direct experience of visiting field sites. This situation is by no means unique to the UK; off-site work often receives less scrutiny and oversight than laboratory-based research in other countries [64], and some have argued that communication and understanding could be facilitated by IACUC vets visiting field research sites in the USA [33,53]. For their part, vets sometimes also acknowledged the value and importance of field visits, with former HOI Craig observing that through field visits ‘[y]ou develop a relationship with them [the researchers]’ (25 June 2019). However, because building these relationships may take time, changes of inspectors (which several participants observed has become more common in recent years) can be disruptive. Researcher Evan, for example, explained that it is

frustrating when inspectors change because you build up a relationship, but you also I think build up knowledge with your inspector. They learn to know what you're doing with the kind of rather strange animals that we sometimes work with, and then you're back to square one with a new one. (15 January 2019)

Alongside visits to the field, and trust gained from long-term relationships, researchers and vets alike highlighted two additional important features of good working relationships: flexibility and mutual respect. The A(SP)A and its associated guidance already contain a certain degree of flexibility, as illustrated by guidance allowing field researchers to use non-standard euthanasia methods in emergencies (see §4). Researchers and vets also often spoke of the importance of both vets and researchers employing ‘flexibility’ or ‘common sense’ in applying and implementing the law. For instance, HOI Gail observed that it may be possible to negotiate compromises for simultaneously minimizing research animal suffering and ecosystem perturbation. Gail described how in a case when sick fish were shedding bacteria, a compromise involved euthanizing unwell fish but putting their bodies back into the water to mitigate the ecological impacts of removing them from the population. Thus, good care in practice—for animal research subjects and ecosystems—may differ to, or go beyond, formal laws and regulations [25,26,28], highlighting the difficulty of creating universally applicable rules about good animal care across contexts [27].

Mutual respect was also highlighted as an important feature of a positive vet–researcher relationship. HOIs and NVSs very often acknowledged researchers as the greatest experts on their study subject and species, and some also noted that they themselves lacked the expertise of wildlife research compared with laboratory work. Former HOI Craig, for example, noted that

if I went to a laboratory animal facility as a Home Office inspector, I felt I was quite comfortable and I was in my environment. If I was out at a POLE then I felt I was probably more in the hands of the researchers in terms of, you know, their views in terms of doing things.

Yet vets also argued that they could make important contributions, in particular because the greater isolation of field researchers from research support communities can impede the updating of animal welfare practices (interview with former HOI Heather, 17 January 2019). Vets also felt that they can make valuable contributions by drawing on their general, non-species-specific veterinary knowledge (interview with NVS Grace, 28 January 2019) and by asking intelligent questions that prompt researchers to reflect on their practices. For example, NVS Elaine argued that to build constructive and effective relationships with researchers, vets must avoid saying, ‘“I know best; do what I say’’. And actually, you need to come from, […] ‘‘You are the expert here. I'm going to ask you lots of questions, so that I can reflect on whether what you're suggesting makes sense”’ (interview, 11 January 2019). For their part, some researchers described finding such constructive questioning and support invaluable. Hugh, for example, noted that both his NVS and HOI acknowledge and respect his species-specific expertise, but he appreciates the ‘challenge’ presented by having someone around to ask intelligent questions (field notes, 29 October 2018).

## 7. Conclusion and recommendations

As we have demonstrated, invasive research with free-ranging animals involves a range of different social, ethical and regulatory challenges compared with laboratory research. While not all of these challenges are resolvable, our qualitative research suggests that flexibility—in policy, personal relationships and animal care practices—and mutual respect between researchers, stakeholders and regulators are important aspirations, which when achieved can help ensure that research with free-ranging animals supports positive outcomes for ecosystems and animals used in research.

### (a) Flexibility

The first key lesson from our qualitative research is that flexibility can play an important role in managing social, ethical and regulatory challenges encountered in field-based animal research. The ethical challenges presented in fields may be perceived as different to those in laboratories, due for example to a lack of control over animals' experiences, and the risk of perturbation. Participants expressed the idea that UK animal research law was not written with these unique challenges in mind and is therefore in some respects an awkward fit for field research. Similar views may arise in other countries, such as the USA. However, there are signs that in the UK this is changing through the development of field-specific guidelines, which was perceived as a positive development [49]. Thus, flexibility can be demonstrated at the level of regulation itself through the development and dissemination of field-specific advice. Furthermore, the A(SP)A was sometimes described as more flexible than other laws affecting wildlife, and that its flexibility is one of its greatest strengths as it enables regulators and researchers to modify animal care practices to best suit the context, and to navigate tensions between individual animal welfare and risks to populations and ecosystems. Participants additionally highlighted the importance of flexibility at a personal level in how researchers, regulators and stakeholders collectively interpret and apply regulation and guidance when managing animal care and ethical issues. These messages highlight how good care in practice in wildlife research—for individual animals, and for broader populations and ecosystems—requires being flexible (see [25,26,28] for related arguments). We therefore recommend that both researchers and other stakeholders working in field-based animal research, rather than sticking to universally applied rules, remain flexible in their approach to applying regulations and guidelines and recognize this may involve going above and beyond legal requirements.

### (b) Mutual respect

The second key theme highlighted by our qualitative research is the importance of mutual respect in interactions between researchers, stakeholders and regulators. Stakeholders (e.g. landowners, anglers, farmers, citizen scientists and indigenous communities in many countries) may play a crucial role in enabling research to proceed. They can also, if brought on board, bring valuable skills (e.g. experience capturing and handling the study species) and knowledge (e.g. location of study species in the local area) that can facilitate and improve research and the experiences of research animals. As well as seeking to make research appealing and valuable to stakeholders—thereby helping to make science more publicly accountable—acknowledging and drawing on stakeholders’ expertise could therefore serve as an important strategy for building positive relationships and promoting good care for animal welfare and ecosystems. Such a move also resonates with the broader shift towards Mode-2 knowledge production and participatory research [21,22].

Similar lessons apply in relationships with regulators. While vets involved in regulatory oversight may lack expertise on field conditions and the species under study, they can develop this knowledge over time, especially via field visits. Furthermore, they can make valuable contributions to research practices and animal care from their more general expertise and ability to ask challenging and intelligent questions of researchers. Participants highlighted the importance of researchers recognizing the importance of vets' contributions, and in turn the need for vets to recognize the species- and field-specific expertise of researchers.

In some ways, flexibility and mutual respect must go together. If, for example, regulators employ flexibility in implementing the A(SP)A but are not respected by researchers, researchers may feel that regulators’ recommendations and decisions are arbitrary (see [65]). Furthermore, both flexibility and mutual respect are to some extent products of long-term relationships and efforts to understand each other's points of view. An important take-home message from our research is therefore that long-term relationships, and other tools for building understanding such as field visits by regulators, should be fostered where possible. However, these steps may be out of the hands of anyone directly involved in research; for example, it may be dictated by institutional policy. There are other factors further limiting the extent to which flexibility and mutual respect can be built. For example, parties involved in research may disagree about fundamental ethical principles, although these disagreements may subside where people make concerted efforts to understand each other's perspectives or reach a mutually acceptable compromise. Where possible we therefore recommended that steps be taken by all those involved in field-based animal research to acknowledge and respect contrary ethical perspectives, in the hope that doing so will benefit the animals used in research, and the populations and ecosystems of which they are part. Furthermore, such efforts to foster mutual respect between researchers and other stakeholders form a key dimension of the ‘culture of care’ in animal research, with implications for the well-being of both the humans and animals involved [66].

## Data accessibility

By agreement with the Wellcome Trust and research participants, anonymised interview transcripts will be deposited in the UK Data Archive based at the University of Essex (https://www.data-archive.ac.uk) after a period of 10 years from the completion of the Animal Research Nexus Project in 2022, except in cases where participants have explicitly opted out of this arrangement. Due to the sensitive nature of the material, data are unable to be released prior to this.
